# Percutaneous Reduction and Fixation with Kirschner Wires versus Open Reduction Internal Fixation for the Management of Calcaneal Fractures: A Meta-Analysis

**DOI:** 10.1038/srep30480

**Published:** 2016-07-26

**Authors:** Jianbin Wu, Feiya Zhou, Lei Yang, Jun Tan

**Affiliations:** 1 Department of Orthopaedic Surgery, the 2nd Affiliated Hospital & Yuying Children’s Hospital of Wenzhou Medical University, Zhejiang Province, China

## Abstract

The aim of our meta-analysis was to compare outcomes for two surgical treatments of calcaneal fractures, percutaneous reduction and fixation with Kirschner wires (PRFK) and open reduction internal fixation (ORIF), with the intent of evaluating the quality of evidence to inform practice. Search of MEDLINE, Cochrane and CNKI databases to identify randomized controlled trials (RCTs) comparing PRKF and ORIF on the following outcomes: post-operative function, complications and quality of the reduction. Odd ratios (OR) and weighted mean differences were pooled using either a fixed-effects or random-effects model, depending on the heterogeneity of the trials included in the analysis. Eighteen RCTs provided the data from 1407 patients. PRFK was associated with a lower risk of surgical wound complications, and ORIF with better post-operative function, angle of Gissane, calcaneal height, and calcaneal width. There were no statistically significant differences between the techniques with regards to post-operative Böhler’s angle. PRFK does not provide a substantive advantage over ORIF for the treatment of calcaneal fractures in adults. PRFK may, however, yield comparable functional outcomes to ORIF for closed Sanders type II calcaneal fractures but with less complication related to surgical wound healing.

Knowledge and expertise in managing closed fractures of the calcaneus in adults has significantly increased over the past few years. Conservative management methods have been popular^1^, consisting of reducing the width of the calcaneus with use of a hammer, taking care to limit soft tissue trauma, followed by plantarflexion of the forefoot to restore the plantar arch. As this technique does not directly reduce the subtalar joint and requires immobilization in a plaster cast, post-traumatic osteoarthritis and joint stiffness are common complications of a conservative management^2^. With advancements in surgical techniques, open reduction and internal fixation with plates (ORIF), via an L-shaped lateral approach, has become the standard method for treating displaced, intra-articular calcaneal fractures^3^. ORIF provides a direct reduction of the articular facet of the calcaneus, which forms the subtalar joint, and allows early mobilization. However, this open reduction technique is associated with a high risk of soft tissue complications related to the surgical wound, such as: hematoma formation, skin edge necrosis, wound breakdown, and superficial or deep infection^4^. Recently, minimally invasive reduction and fixation has emerged as an alternative to ORIF to avoid soft tissue complications^5^. In recent years, the popularity of using percutaneous reduction and fixation with Kirschner wires (PRFK) as a minimally invasive treatment for calcaneal fractures has increased in China. This procedure usually consists of inserting a Kirschner wires (K-wire), using a joystick, from the calcaneal tuberosity to beneath the subtalar joint, with plantarflexion of the forefoot used to reduce the fracture. After the K-wire is advanced into the distal fracture fragment, augmentation fixation using one or two K-wires is necessary. However, there are no clear guidelines to inform the selection of either a PRFK or ORIF approach for the management of calcaneal fractures in adults. Therefore, we conducted a literature search to identify randomized controlled trials (RCTs) comparing PRFK and ORIF techniques and performed a meta-analysis with the intent of evaluating the evidence to inform selection of the preferred technique.

## Materials and Methods

### Search strategy

A search of MEDLINE, Cochrane and CNKI databases was performed from their inception to November 2015, without limitations with regards to study design, using the following MeSH (Medical Subject Heading) terms and text words in different combinations: calcaneus, subtalar joint, fractures, calcaneus fractures, calcaneus fracture, calcis fractures, and calcis fracture. These subject-specific terms were combined with the Cochrane Highly Sensitive Search Strategy, sensitivity- and precision-maximizing version, to identify RCTs^6^. The search was supplemented by a manual citation search of the reference lists of relevant studies identified.

### Inclusion Criteria/Exclusion Criteria

Only RCTs were included in our meta-analysis, with quasi-randomized trials and non-randomized trials excluded. All RCTs comparing PRFK to ORIF for closed fractures of the calcaneus in patients over the age of 18 years, treated in an acute setting within 3 weeks of the injury, were eligible. The technique of PRFK is described in the introduction. The procedure of ORIF consists of open reduction via L-shaped lateral approach and internal fixation with plate.

### Outcomes of Interest

The following post-operative outcomes of interest were included in the analysis: functional status; complications related to the surgical wound, such as infection, hematoma formation, wound dehiscence, surgical wound edge necrosis, and sural nerve injuries; long term complications, such as post-traumatic osteoarthritis, heel pain, and tendinitis (the follow-up duration should be beyond 2 years); and quality of the reduction.

### Study Selection and Data Extraction

Two reviewers independently assessed the eligibility of identified RCTs in an unblinded standardized manner. A data extraction sheet was developed based on the Cochrane Consumers and Communication Review Group’s data extraction template^7^. Data were collected independently by the two reviewers and disagreements resolved by discussion with a senior author. The following information was extracted from the RCTs: research method; characteristics of trial participants, including age, fracture classification, and smoking status; the trial’s inclusion and exclusion criteria; interventions characteristics, including surgical approach and fixation method used; post-operative outcomes of interest; and risk of bias. When information was missing, we attempted to contact the primary author by email to seek clarification.

### Quality Assessment

The risk of bias was evaluated independently by two of the review authors using the domain-based evaluation described in the Cochrane Handbook for Systematic Reviews of Interventions^8^. The following domains were assessed: random sequence generation; allocation concealment; blinding of participants, research personnel and outcome assessors; incomplete outcome data; and selective outcome reporting. Each of these criteria was assessed as ‘low risk of bias’, ‘high risk of bias’ or ‘unclear risk of bias’, when there was lack of information or uncertainty over the potential for bias. The quality of the evidence was quantified using the GRADE approach^9^, with disagreements between the review authors regarding the risk of bias for the identified domains resolved by consensus.

### Statistical Analysis

The meta-analyses were performed using the Review Manager software (RevMan Version 5.3; The Nordic Cochrane Center, Denmark). In addition, tests for funnel plot asymmetry were performed with Stata software (StataSE 12.0; StataCorp LP, USA). Odd ratios (ORs), together with 95% confidence intervals (CIs), were calculated for dichotomous outcomes. Continuous outcomes were expressed as mean differences (MDs), with corresponding 95% CIs. The standardized mean difference (SMD) was calculated when the same outcome was measured by different scales in different trials, or the same outcome was reported by both dichotomous and continuous data. Heterogeneity across trials was tested by chi-squared analysis, with the I^2^ statistic used to assess the impact of identified heterogeneity on the meta-analysis. Substantial heterogeneity was defined as an I^2^ > 50%. If substantial heterogeneity between trials included in an analysis was identified, estimates of pooled data were evaluated using a random-effect model; otherwise, a fixed-effect model was chosen. Subgroup analysis was conducted to investigate the influence of differences in the fracture type on pooled estimates. Funnel plot asymmetry was assessed using Begg and Egger tests.

## Results

### Search Results

**Figure 1:**
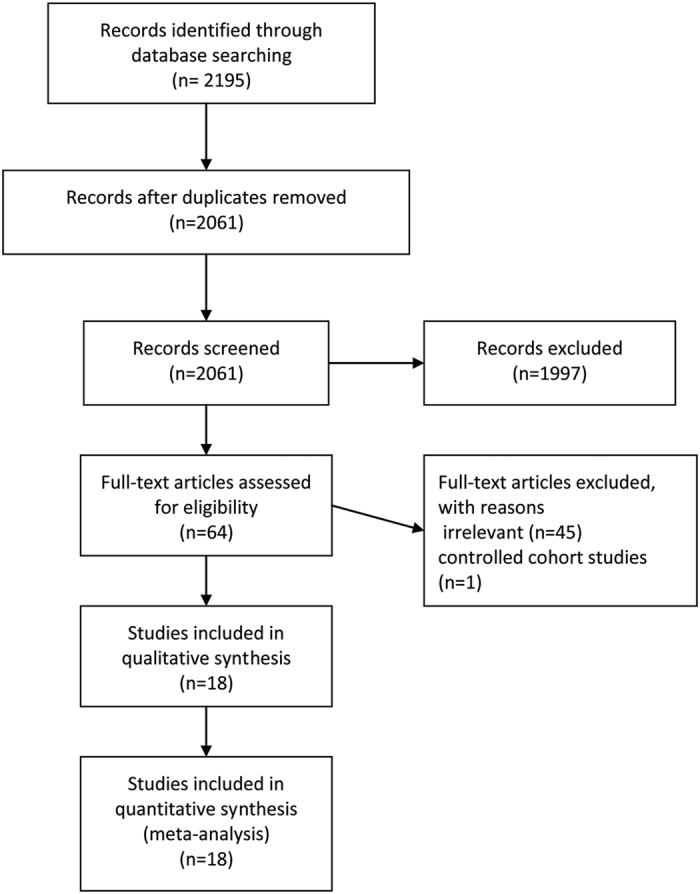
Flow Diagram of Search.

The details of our search strategy and exclusion criteria are presented in the flow diagram in Fig. 1. A total of 2195 titles and abstracts were identified and screened, with 18 trials satisfying our eligibility criteria^10^,^11^,^12^,^13^,^14^,^15^,^16^,^17^,^18^,^19^,^20^,^21^,^22^,^23^,^24^,^25^,^26^,^27^. All of the included trials were RCTs, comparing PRFK and ORIF in the treatment of calcaneal fractures in adults.

### Quality Assessment

**Figure 2:**
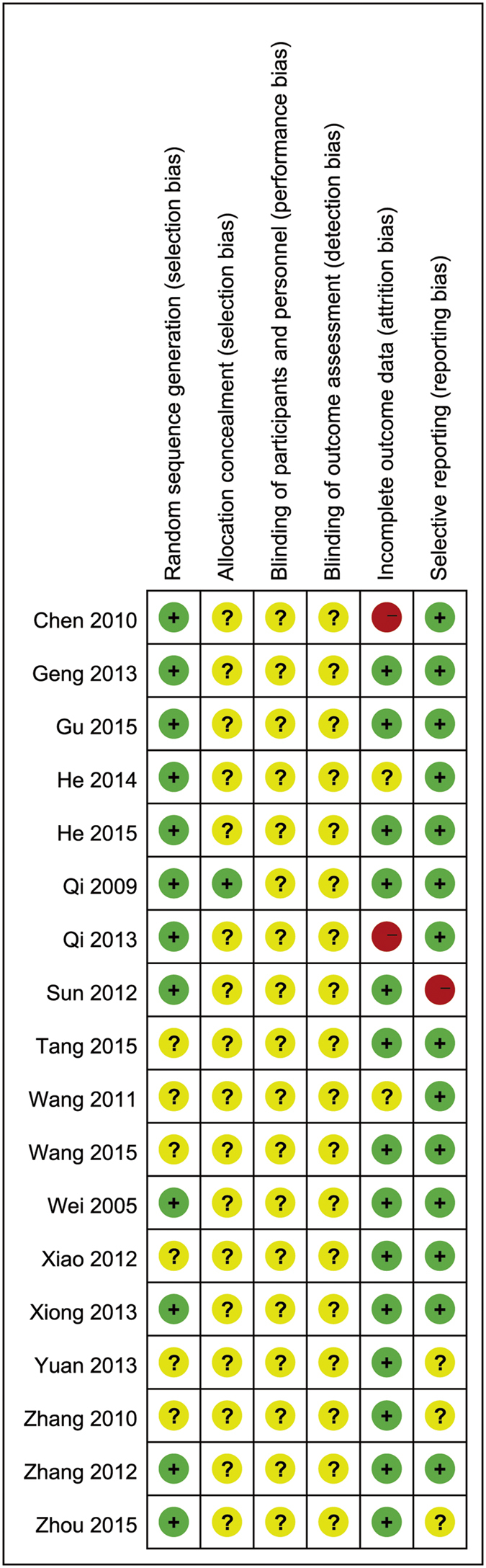
Summary of Risk Bias Assessment. Note: Reviewers’ assessment of each risk of bias item; “+”, low risk of bias; “?”, unclear risk of bias; and “−”, high risk of bias.

**Figure 3:**
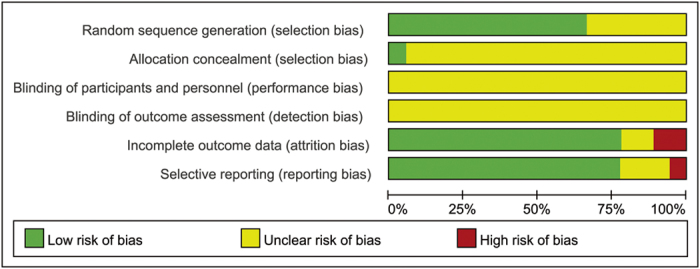
Risk of Bias Graph. Note: Reviewers’ assessment of each risk bias item, presented as a percent across all included RCTs.

The risk of bias was moderate for all included studies (see Figs 2 and 3). Adequate randomization method was reported in 12 trials; lottery method^17^, table of random number^10^,^13^,^14^,^15^,^18^,^19^,^22^,^24^,^26^,^27^ and computer-generated number^20^ methods were used. The method of randomization was not reported in the other 6 trials^11^,^12^,^16^,^21^,^23^,^25^. Only one trial described the method of allocation concealment^15^. Although no information of method of blinding was included in the trials, we assumed that participants, research personnel and outcome assessors had full knowledge of the surgical technique used and, hence, of group assignment.

### Descriptive Characteristics

The descriptive characteristics of the included studies are listed in Table 1, with relevant characteristics summarized here. All the included RCTs were reported in Chinese, and all were single-center trials performed in China. All the studies were parallel randomized controlled trials, with two intervention groups. Open fractures were excluded in all 18 trials.

Together, identified RCTs enrolled a total of 1426 patients. After accounting for participants lost to follow-up, data from 1407 participants, providing 1442 fractures, were entered into our meta-analysis. Males accounted for 72.4% of participants. The type of fracture, assessed using Sander’s classification of calcaneal fractures, varied across trials, with 4 RCTs including only Sander’s type II fractures^15^,^16^,^17^,^18^, 5 including Sander’s type II and III fractures^19^,^20^,^21^,^22^,^23^, 3 RCTs including Sander’s type II, III and IV fractures^12^,^13^,^14^, and two including Sander’s type I, II, III and IV fractures^10^,^11^; the fracture type was not reported in the other 4 RCTs^24^,^25^,^26^,^27^. None of the included studies described the smoking status of participants. All of the included studies described the technique of PRFK as mentioned previously. The K-wires were removed in 8–10 weeks.

### Effects of Interventions

#### Functional Outcome

**Figure 4:**
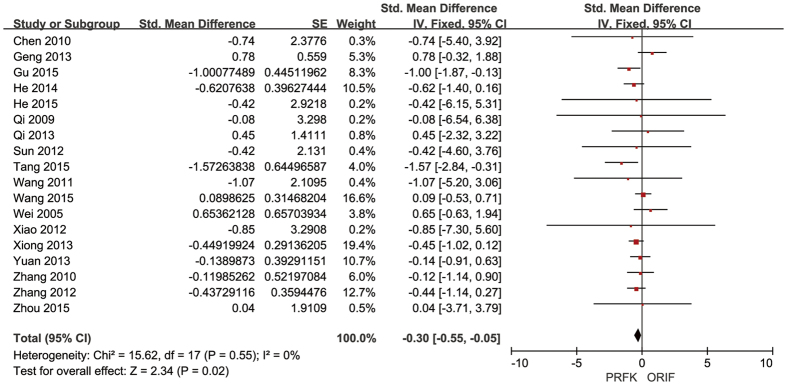
Forest Plot of SMDs and Associated 95% Confidence Intervals for Functional Outcomes.

Different scales were used across trials to assess and report patients’ functional scores: 13 trials reported the Maryland Foot Score (MFS)^11^,^12^,^13^,^15^,^16^,^18^,^19^,^21^,^22^,^23^,^25^,^26^,^27^; one trial the Kerr-Atkin’s score^20^; one the American Orthopaedic Foot & Ankle Society score (AOFAS)^17^; and one the functional estimate scale of Tu Chongqi^12^. The functional estimate scale was not identified in the other two trials^14^,^24^. Pooled data, shown in Fig. 4, indicated better post-operative functional outcomes for patients treated by ORIF, compared to those treated by PRFK (SMD = −0.30, 95% CI, −0.55–−0.05; P = 0.02).

#### Complications

**Figure 5:**
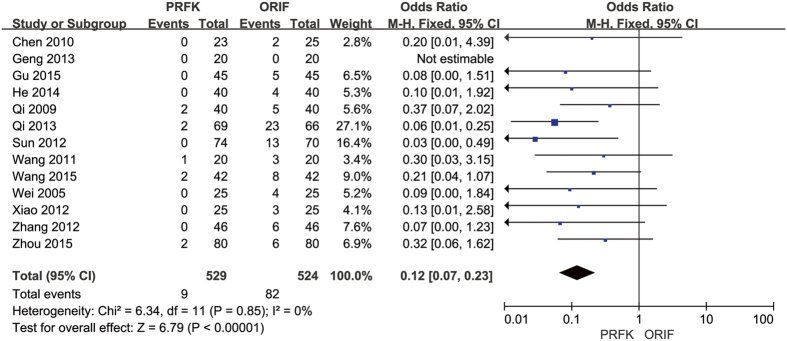
Forest Plot of OR, and Associated Confidence Intervals, for Complications Related to the Surgical Wound.

Complications related to the surgical wound were reported in 13 trials^13^,^14^,^15^,^16^,^17^,^18^,^19^,^20^,^21^,^22^,^23^,^24^,^27^, with a calculated OR of 0.12 (95% CI, 0.07–0.23; P < 0.00001; Fig. 5). Four trials reported complications, such as post-traumatic osteoarthritis, heel pain, and tendinitis. However, it is important to note that none of the follow-up periods extended beyond 2 years.

#### Quality of the Reduction

**Figure 6:**
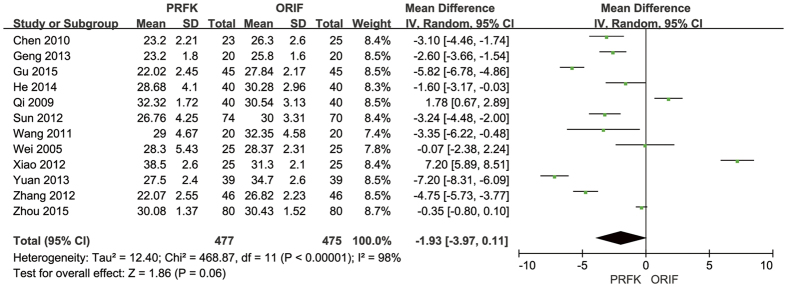
Forest Plot of MD, and Associated Confidence Intervals, for the Angle of Böhler’s.

**Figure 7:**
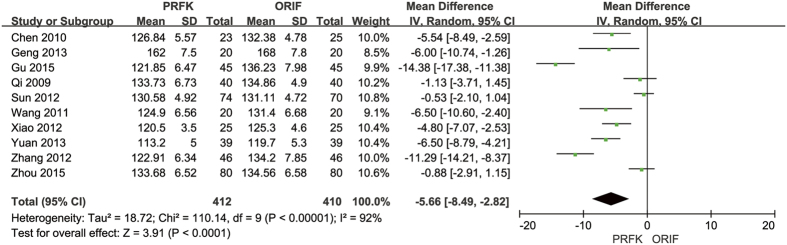
Forest Plot of MD, and Associated Confidence Intervals, for the Angle of Gissane.

The post-operative Böhler’s angle was reported in 12 trials^11^,^13^,^14^,^15^,^16^,^17^,^18^,^19^,^21^,^22^,^24^,^27^, with analysis of the pooled data, shown in Fig. 6, indicating a MDs of −1.93° (95% CI, −3.97–0.11; P = 0.06). The post-operative angle of Gissane was reported in 10 trials^11^,^13^,^14^,^15^,^16^,^17^,^18^,^19^,^21^,^22^, with analysis of the pooled data, shown in Fig. 7, indicating a MD of −5.66° (95% CI, −8.49–−2.82; P < 0.0001).

**Figure 8:**
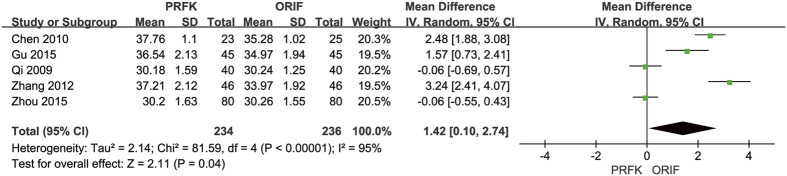
Forest Plot of MD, and Associated Confidence Intervals, for Calcaneal Widths.

**Figure 9:**
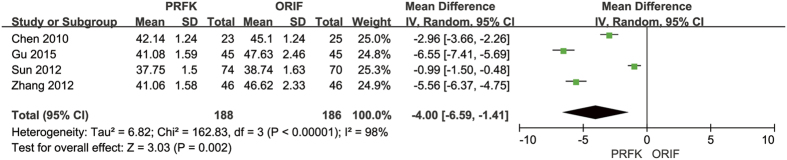
Forest Plot of MD, and Associated Confidence Intervals, for Calcaneal Height.

In terms of calcaneal width, 5 trials reported their post-operative calcaneal widths^13^,^14^,^15^,^18^,^22^; the pooled data shown in Fig. 8, with a calculated MD of 1.42 mm (95% CI, 0.10–2.74 mm; P = 0.04). Post-operative calcaneal height was reported in 4 trials^13^,^14^,^19^,^22^, with the pooled data shown in Fig. 9, indicating a MD of −4.00 mm (95% CI, −6.59–−1.41 mm; P = 0.002).

### Publication Bias

An assessment of publication bias was conducted for the factors of functional outcomes for the overall population. The analysis did not identify any potential publication bias (Egger test, P = 0.917; Begg test, P = 1.00).

### Subgroup Analysis

Of the trials included in the meta-analysis, 4 trials reported outcomes for Sander’s type II fractures^15^,^16^,^17^,^18^ and two trials for Sander’s type II and III fractures, separately^19^,^20^. Therefore, we performed a subgroup analysis of Sander’s type II fractures, with regards to functional outcome, Böhler’s angle, the angle of Gissane, calcaneal width, and complications related to surgical wounds. For the angle of Gissane, the analysis favored outcomes with ORIF compared to PRFK (MD = −2.25°; 95% CI, −4.26–−0.24; P = 0.03). Outcomes were comparable for ORIF and PRFK with regards to Böhler’s angle (MD = −0.81°; 95% CI, −2.30–0.69; P = 0.29), calcaneal width (MD = −0.06 mm; 95% CI, −0.45–0.33; P = 0.76) and functional outcome (SMD = 0.56; 95% CI, −0.39–1.51; P = 0.25). The risk for complications related to surgical wounds was lower for PRFK than for ORIF (OR = 0.24; 95% CI, 0.09–0.63; P = 0.004).

## Discussion

Calcaneal fractures are frequent fractures managed in orthopedic clinics. Although ORIF is commonly used to treat these fractures, a recent meta-analysis stated that, compared to non-operative treatment, ORIF could reduce the risk of late subtalar arthrodesis, but with a significantly higher risk of complications^28^. A variety of minimally invasive reduction and fixation methods have emerged in recent years, providing an alternative to ORIF^4^. These emergent techniques include: percutaneous reduction and internal or external fixation; reduction through a minimally invasive approach with internal or external fixation; and arthroscopic-assisted reduction with internal or external fixation. We conducted a meta-analysis to compare and contrast outcomes of two surgical techniques, percutaneous reduction and fixation with K-wires (PRKF), and open reduction internal fixation with plates (ORIF), via an L-shaped lateral approach, to provide evidence necessary to guide clinical decisions on the selection of the most appropriate technique to treat closed fractures of the calcaneus in adults.

PRFK was associated with a lower risk of complications related to surgical wounds, compared with ORIF. However, patients treated by ORIF achieved better post-operative functional outcomes. ORIF also yielded better outcomes with regards to selected parameters of the quality of reduction, namely the angle of Gissane, calcaneal height and calcaneal width, with no treatment advantage on Böhler’s angle.

The trials included outcomes for different Sander’s types of calcaneal fractures. Therefore, pooled estimates should be interpreted with caution. To address this heterogeneity across trials, we performed a subgroup analysis comparing outcomes of PRFK and ORIF specifically for Sander’s type II fractures. The subgroup analysis did not identify significant differences between the two methods in terms of post-operative functional outcomes, Böhler’s angle and calcaneal width. The ORIF was associated with a better angle of Gissane. Our subanalysis, however, did identify a lower risk for complications related to surgical wounds with PRFK. Across the trials included in our analysis, reporting of calcaneal height was limited and, therefore, we did not perform a subgroup analysis with regards to this variable.

Although our results indicates that ORIF can achieve better post-operative Gissane’s angle, both in general pooled data and subgroup analysis, this finding should be interpreted with caution. William Gissane first described the crucial angle in 1947^29^, which later was called “Gissane’s angle”, “crucial angle of Gissane” or “critical angle of Gissane”. We could not obtain the original description of Gissane’s angle. In his article, Essex-Lopresti defined Gissane’s angle as the angled strut supporting the sharp lateral spur of the talus^30^. In several papers, Gissane’s angle is formed by the posterior facet and line from the sulcus calcaneus to the tip of the anterior process^31^,^32^. According to the mechanism of the calcaneus fractures, the Gissane’s angle will be disrupted by the primary fracture line^30^. Theoretically, restoration and maintenance of Gissane’s angle is necessary for satisfactory results after calcaneus fractures. Although a number of researches have used Gissane’s angle to evaluate radiological reduction, some studies failed to demonstrate the prognostic value of Gissane’s angle^33^,^34^,^35^. Although Persson implied that worse Gissane’s angle correlated with worse functional outcomes, the effect size of this correlation was not statistically significant^36^.

All 18 trials included in our meta-analysis were RCTs, with 12 trials assessed to be at low risk for selection bias due to adherence to strict randomization technique^10^,^13^,^14^,^15^,^17^,^18^,^19^,^20^,^22^,^24^,^26^,^27^. The allocation concealment was not adequate. As the surgical technique could not be concealed from participants, personnel and assessors, blinding could not be feasibly achieved. Due to lack of allocation concealment and blinding of participants, personnel and outcome assessment, we conclude that the quality of evidence for functional outcome to be of moderate quality. With the added effect of high heterogeneity, we also concluded the quality of evidence for Böhler’s angle and the angle of Gissane to be low. With the aforementioned reasons and the low recruitment, we concluded the quality of evidence for calcaneal height and calcaneal width to be very low. In contrast, while lack of allocation concealment and blinding was also a problem for complications related to surgical wound, due to the large estimates of effects, we conclude the quality of evidence for complications related to surgical wound to be high.

We have searched three data base, the search strategy and research protocol was strictly complied with the PRISMA statement for reporting systematic reviews and meta-analysis. The two intervention groups in the included studies, namely PRFK and ORIF, were highly coincident. Still, there are several limitations in our meta-analysis. First, all of the included studies were small, single center trials conducted in China, and they were all published in Chinese. Second, baseline characteristics of patients, as well as inclusion and exclusion criteria varied among the included trials. The heterogeneity in patient characteristics across trials is important to consider as clinical and radiological outcomes could be affected by confounding factors, such as fractures type, patients’ age, surgeons’ experience, and presence of other patient comorbidities. Third, the follow up period of the trials included was short to moderate and, therefore, long-term complications, such as post-traumatic osteoarthritis which might take years to develop, could not be evaluated. Fourth, none of the included studies described the smoking status of the patients, which is critical to surgical wound healing and fracture union.

In summary, our study is the first meta-analysis to compare outcomes of PRFK and ORIF for the treatment of calcaneal fractures in adults. Based on evidence evaluated, PRFK does not provide a substantive advantage over ORIF, except in lowering the risk for complications related to the surgical wounds. Our subgroup analysis of outcomes specifically for Sander’s type II calcaneal fractures confirmed comparable functional outcomes for both procedures, with a significantly lower risk for complications related to surgical wounds with PKRF. Multicenter RCTs, with high methodological quality and long term follow-up period are needed. Based on current evidence, treatment decision must be made based on patients’ characteristics, fracture morphology, soft tissue status, and surgeons’ experience.

## Additional Information

**How to cite this article**: Wu, J. *et al*. Percutaneous Reduction and Fixation with Kirschner Wires versus Open Reduction Internal Fixation for the Management of Calcaneal Fractures: A Meta-Analysis. *Sci. Rep.* **6**, 30480; doi: 10.1038/srep30480 (2016).

## Figures and Tables

**Table 1:** Descriptive Characteristics of Included Trials.

Study	Year	Sample size(PRFK) (P/F)	Sample size(ORIF) (P/F)	Age(PRFK)	Age(ORIF)	Sex (M/F)(PRFK)	Sex (M/F)(ORIF)	Functional Scale	Follow-up (mo)	Lost to Follow-up	Sanders fracture classification
Xiong	2013	20/20	20/20	43.7 ± 2.4	43.1 ± 1.5	16/4	17/3	MFS	17 (8–38)	0	Type I, II, III, and IV
Yuan	2013	39/39	39/39	38.3 ± 4.1	40.0 ± 3.9	31/8	33/6	MFS	N/A	0	Type I, II, III, and IV
Zhang	2010	*	*	*	*	*	*	TCQ	3–24	0	Type II, III, and IV
Zhang	2012	46/46	46/46	37.5 ± 4.8	38.2 ± 5.1	37/9	35/11	MFS	12	0	Type II, III, and IV
Gu	2015	45/45	45/45	36.5 ± 4.7	38.3 ± 5.2	32/13	29/16	N/A	12	0	Type II, III, and IV
Qi	2009	40/40	40/40	36.6 ± 3.2	37.1 ± 3.4	26/14	30/10	MFS	12	0	Type II
Wang	2011	20/20	20/20	N/A	N/A	15/5	15/5	MFS	12	0	Type II
Geng	2013	20/20	20/20	40.1 ± 4.8	43.3 ± 2.1	15/5	16/4	AOFAS	6	0	Type II
Zhou	2015	80/80	80/80	**	**	**	**	MFS	12	0	Type II
Sun	2012	74/74	70/70	20–59	20–59	53/21	55/15	MFS	12 (9–15)	9	Type II, and III
Qi	2013	69/82	66/75	18–64	21–63	60/9	58/8	Kerr	6	8	Type II, and III
Xiao	2012	25/25	25/25	47.5 ± 4.7	47.3/5.8	22/3	20/5	MFS	9 (6–13)	0	Type II, and III
Chen	2010	25/25	22/25	39.2 ± 8.4	38.9 ± 7.8	14/11	17/8	MFS	12	0	Type II, and III
Wang	2015	42/42	42/42	38.4 ± 1.5	38.2 ± 1.2	20/22	21/21	MFS	N/A	0	Type II, and III
Wei	2005	20/20	20/20	20–61	20–61	18/2	19/1	N/A	10 (8–12)	0	N/A
Tang	2015	13/14	12/14	20–63	20–68	12/1	12/0	MFS	N/A	0	N/A
He	2015	48/48	48/48	54.1 ± 12	53.8 ± 12	31/17	29/19	MFS	N/A	0	N/A
He	2014	40/48	40/40	21–45	18–51	38/10	41/7	MFS	12	16	N/A

Notes: PRFK, percutaneous reduction and fixation with Kirschner wires; ORIF, open reduction and internal fixation with plates via L-shaped lateral approach; P/F, participants/fractures; M/F, male/female; MO, month; MFS, Maryland Foot Score; TCQ, the functional estimate scale of Tu Chongqi; AOFAS, American Orthopaedic Foot & Ankle Society score; Kerr, Kerr-Atkins score; *, a total of 59 participants, with 38 fractures in the PRFK group and 31 fractures in ORIF group, a mean age of 38.5 years, and a M/F ratio of 40/19; **, a mean age of 38.1 ± 3.5 years and a M/F ratio of 102/58.
